# Advancements and prospects in key technologies for robotic pollination in greenhouse pepper breeding: a review

**DOI:** 10.3389/fpls.2026.1778541

**Published:** 2026-02-27

**Authors:** Minqiu Kuang, Xiaojian Li, Fangping Xie, Xuejie Zou, Yang Xiang, Yuxuan Zhang, Dawei Liu, Xiangjun Zou, Xu Li

**Affiliations:** 1 School of Mechanical and Electrical Engineering, Hunan Agricultural University, Changsha, China; 2 Foshan Zhongke Agricultural Robotics and Smart Agriculture Innovation Institute, Foshan, China; 3 Hunan Provincial Key Laboratory of Intelligent Agricultural Machinery Equipment, Changsha, China; 4 College of Intelligent Science and Engineering, Beijing University of Agriculture, Beijing, China; 5 Department of Computer and Electrical Engineering, Mid Sweden University, Sundsvall, Sweden

**Keywords:** artificial intelligence, breeding, end effector, path planning, pepper, precision pollination, visual perception

## Abstract

Robotic pollination represents a pivotal component of smart agriculture, with foundational architectures for target recognition, path planning, and motion control having been progressively established. However, developing an efficient and robust pollination system that integrates perception, decision-making, and execution within real-world scenarios remains confronted with complex challenges. This study systematically reviews recent advancements in the field and distills the core technical issues of greenhouse robotic pollination into three primary domains: target detection and pose estimation, end-effector design, and pollination strategies combined with motion control. Focusing on the visual perception of flowers, actuator architecture, and operational tactics, this review synthesizes existing academic findings to evaluate the state-of-the-art in flower detection and pose estimation, characterize diverse end-effector designs, and analyze the evolutionary trajectory of motion control techniques. Specifically, the analysis encompasses the impact of detection algorithms on recognition accuracy and robustness, the structural classification and performance attributes of pollination mechanisms, and the optimization of control strategies. Furthermore, the study categorizes global research backgrounds, technical methodologies, and paradigmatic system cases, offering a critical evaluation of experiences in constructing automated pollination systems. Despite these advances, current robotic pollination technologies for peppers (chili) face significant bottlenecks characterized by immature methods for precise flower detection and pose estimation, the need for optimized specialized end-effector designs, and insufficient robustness in decision-making systems under dynamic environmental conditions. To address these issues, future development should prioritize constructing diverse, large-scale flower image and pose datasets while developing detection algorithms adaptable to complex environments to achieve high-precision identification. Additionally, implementing this system requires a hierarchical architecture where perception drives adaptive actuation. Deep learning models must localize flower targets and assess maturity in real-time, feeding coordinates to path planners that generate collision-free trajectories through foliage. These trajectories are executed via multimodal motion control, synchronizing the rigid manipulator with soft end-effectors. By embedding tactile feedback into the machine learning loop, the system creates a unified sensorimotor framework. This enables dynamic force modulation based on physical resistance, ensuring precise, non-destructive pollination tailored to chili plants.

## 1 Introduction

Chili pepper represents a cornerstone of protected agriculture in China, with cultivation spanning a vast geographic expanse from Xinjiang in the west to Shanghai in the east, and extending from Hainan Island in the south to Heilongjiang in the north. As a vegetable crop ranking among the global leaders in both cultivation area and consumption volume (Zou et al., 2020), the production of peppers has evolved significantly through advanced planting techniques and expanded acreage. Modern facilities, including large-scale greenhouses and solar greenhouses, have gained widespread adoption due to their superior economic efficiency compared to open-field farming. These controlled environments optimize plant growth conditions by regulating temperature and humidity, ensuring frost resistance, and providing optimal illumination, thereby enabling continuous, year-round production (Zhao et al., 2023).

In the context of agricultural modernization, seeds are often likened to the “semiconductors” of agriculture; consequently, the quality of pepper seeds is fundamental to enhancing crop quality, augmenting yields, and fostering industrial advancement. Pollination serves as a critical juncture in pepper seed production and fruit development, directly influencing seed formation and overall fruit quality (Lin et al., 2023). Effective pollination significantly improves fruit set rates and ensures that plants produce a higher quantity of superior fruits under identical growth conditions (Tian et al., 2019). However, current pollination practices rely predominantly on manual labor, a method fraught with challenges such as high labor intensity, recruitment difficulties, low efficiency, and inconsistent operation quality (Zhang and Zhang, 2015; Gu et al., 2018). Therefore, investigating mechanized precision pollination technologies and developing specialized equipment for peppers provides essential technical support for the revitalization of the seed industry and addresses core technological bottlenecks in germplasm resources.

In the broader context of solanaceous crops, Zhang et al. (2024) conducted an extensive assessment of precision pollination robotics for greenhouse tomatoes to analyze current advancements and prospective trends. Their work scrutinized target recognition technologies for tomato blossoms and evaluated various end-effector configurations (Zhao et al., 2022). Furthermore, they identified existing obstacles in end-effector research, proposed remedial strategies, and examined the feasibility of liquid spray pollination technology. While this research offers valuable insights for greenhouse tomatoes, chili presents unique requirements. Although peppers are hermaphroditic, self-pollinating crops, they typically rely on wind and insect vectors to facilitate pollen release and transfer in natural settings. In enclosed greenhouse environments, physical barriers such as shade nets and plastic films obstruct these natural pathways, resulting in inadequate pollination that adversely impacts fruit set, yield, and quality (Shen and Hao, 2004; Lin et al., 2017). The rapid expansion of greenhouse farming, compounded by rural labor shortages and an aging agricultural workforce, necessitates the broad implementation of artificial intelligence-driven target identification By facilitating intelligent, automated, and efficient pollination processes, robotic systems mitigate the limitations of manual operation and pave the way for enhanced pepper productivity and quality.

**Figure 1:**
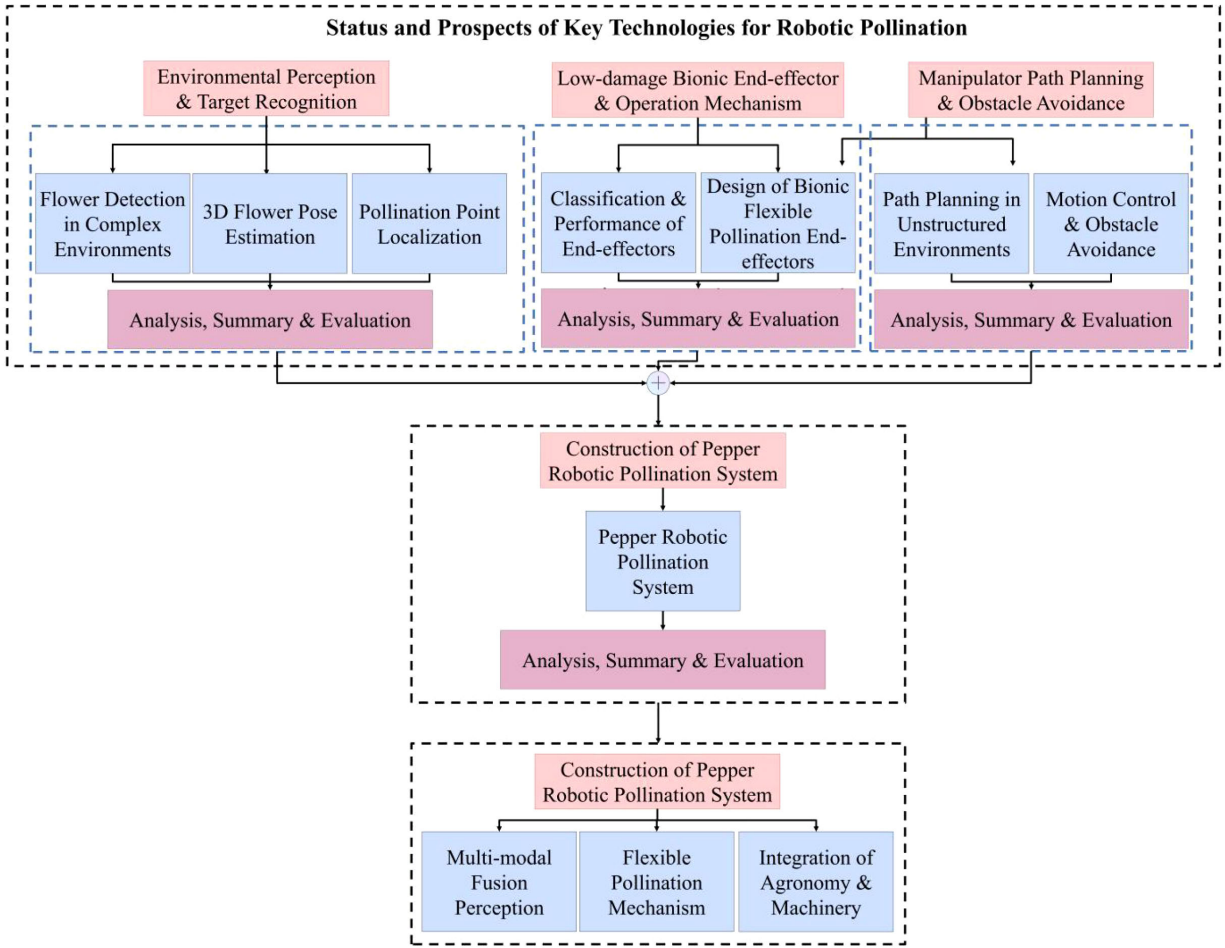
Content organization framework of key mechanical pollination technologies and operational systems.

To provide a comprehensive understanding of the latest advancements and future trends in this field, this paper systematically analyzes essential technologies for the precision pollination of facility fruits and vegetables. This review examines the comparative advantages and limitations of various pollination systems, highlights key challenges specific to facility-based pepper pollination, and proposes focused solutions and innovative strategies. The objective is to serve as a foundational reference for researchers in the sector, thereby advancing the evolution of China’s facility pepper industry toward more intelligent and efficient practices. **Figure 1** illustrates the organizational framework of the key mechanical pollination technologies and operational systems discussed herein.

## 2 Global research advances in flower object detection and pose estimation

**Figure 2:**
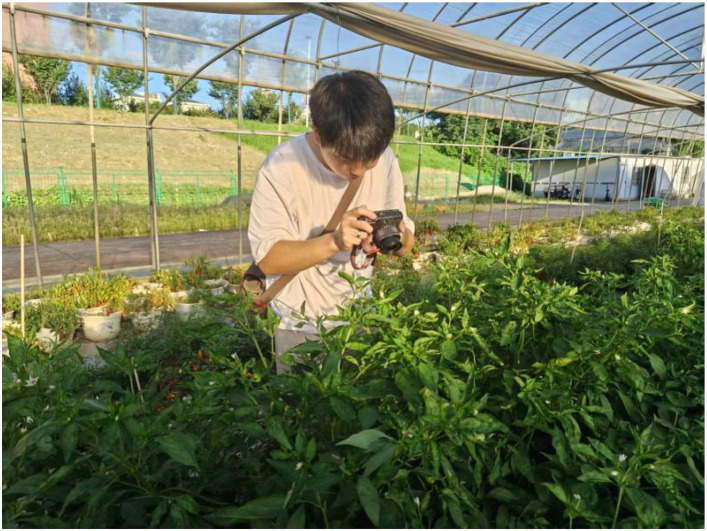
Facility pepper cultivation and data collection scenario.

In the development of precision pollination systems for greenhouse-cultivated peppers, the accurate detection of flowers and the precise assessment of their orientations constitute the primary and most critical prerequisites (Tang et al., 2012). However, achieving this in a greenhouse environment presents substantial challenges for the design and motion control of pollination equipment. These difficulties stem from variable lighting conditions, diverse plant morphologies, and the intrinsic characteristics of pepper flowers, which include their diminutive size, dense distribution, severe occlusion by foliage, overlapping blossoms, and varied orientations. The precision of detection and pose estimation directly dictates the accuracy and operational efficiency of the pollination machinery (Zhang et al., 2023). As illustrated in **Figure 2**, the complex growth environments of greenhouse crops such as peppers and tomatoes are characterized by diverse flower poses and significant occlusion between flowers and leaves. These factors exacerbate the difficulty for visual recognition systems to identify targets swiftly and precisely (Long et al., 2021; Chen and Meng, 2025; Liu et al., 2020; Kuang et al., 2025a; 2025b; 2025c). Consequently, achieving efficient and accurate identification of pepper flowers within facility agriculture environments remains a significant bottleneck that necessitates urgent resolution to propel the advancement of robotic precision pollination technology.

### 2.1 Machine vision-based flower object detection methods

In the nascent stages of flower detection research, methodologies predominantly relied on conventional visual analysis techniques, focusing primarily on chromatic attributes for identification. Image processing techniques, particularly segmentation algorithms, were employed to isolate flower regions from the background based on color patterns and their proportional distribution within the image. However, this approach possesses inherent limitations as it relies exclusively on color as a single dimension. Given the prevalence of similar hues among various floral species in nature, identification based solely on color often lacks precision. To enhance recognition accuracy, it is essential to integrate not only chromatic attributes but also morphological factors, such as flower size, shape, and distinctive textural or structural features. These integrated features establish a comprehensive foundation for recognition, thereby significantly improving the accuracy and robustness of the system.

Several studies illustrate the evolution of these methods. Feng et al. (2013) introduced a machine vision-based technique for acquiring growth data of flower seedlings, employing binary image segmentation to categorize seedlings as absent, substandard, or superior, achieving an accuracy rate exceeding 87%. However, this method struggled with interference between seedlings and cotyledons, leading to misclassification and compromised detection accuracy. Similarly, Dorj et al. (2013) developed a computer vision-based binary method to detect and enumerate citrus blossoms under natural lighting for yield estimation. By employing Gaussian filters to mitigate noise and adjust lighting, the algorithm attained an accuracy of 80.55% across all trees. Although 1,340 sub-images of citrus blooms were identified from 21 trees, the approach remained sensitive to illumination variations and noise, resulting in elevated error rates.

In another approach, Yang et al. (2022) proposed a color template matching system for the non-destructive assessment of Phalaenopsis orchids. By capturing multi-angle images via rotation, the method extracted flower areas to evaluate blooming degree and quality. Despite its intent, the method demonstrated a significant error margin ranging from 0.7% to 64.8% when applied to images from various viewing angles. Furthermore, the requirement to rotate potted plants to capture comprehensive data rendered the detection process complex and limited its practical utility. Conversely, Cui et al. (2019) innovatively employed an improved K-means clustering algorithm for the efficient segmentation of strawberry images. Combined with morphological processing and connected component analysis, this method accurately delineated flower regions. Ohi et al. (2018) proposed a hybrid strategy integrating long-range and short-range detection. The long-range phase utilized cameras for initial localization and color classifiers for coarse segmentation to swiftly identify putative floral patches. The short-range phase employed RGB-D cameras with real-time dense Simultaneous Localization and Mapping (SLAM) technology to reconstruct strawberry plants in 3D, achieving an overall recognition accuracy of 78.6%. Nevertheless, this technique remained vulnerable to interference from similar-looking flowers, resulting in suboptimal overall efficacy.

In summary, conventional machine vision techniques are often characterized by low detection accuracy, inadequate resistance to interference, and insufficient robustness. Conversely, deep learning, particularly the application of Convolutional Neural Networks (CNNs) (Krizhevsky et al., 2012), has significantly enhanced floral recognition accuracy. These algorithms autonomously learn intricate crop characteristics and minimize classification errors while exhibiting remarkable resilience to complex growth conditions, occlusions, and environmental disturbances. Due to advantages in automated feature extraction, precision, robustness, and computational efficiency, deep learning is progressively supplanting traditional machine vision methods in flower recognition applications.

### 2.2 Method of flower object detection and pose estimation based on machine learning

In contrast to conventional machine vision algorithms, machine learning-based solutions for flower object detection and pose estimation more accurately replicate human recognition patterns. CNNs serve as a pivotal technology for extracting high-level features, enabling the direct derivation of sophisticated semantic information from raw images. This end-to-end perception process obviates the need for laborious manual feature engineering. Consequently, neural networks exhibit remarkable adaptability to intricate and dynamic environments, representing the future direction for developing robust and intelligent vision systems (Paul et al., 2025). Recent advancements in high-performance computing have further accelerated the deployment of large-scale neural network algorithms on robotic arms, dramatically enhancing their efficacy in object detection and pose estimation. Significant milestones have been achieved in domains including fruit and vegetable flower recognition, spatial localization, and complex phenotypic reconstruction (Paul et al., 2025; Xiong et al., 2025; Huang et al., 2023; Kamata et al., 2018).

Regarding flower object detection, deep learning techniques exhibit superior robustness and adaptability compared to traditional methods based on color difference, color space, or clustering (Xie et al., 2019; Qiu et al., 2022). They successfully surmount obstacles such as vegetation occlusion and lighting fluctuations, thereby surpassing traditional methods in both recognition precision and processing efficiency. Leveraging the end-to-end nature of deep learning streamlines the design and execution of recognition algorithms, which improves deployment efficiency (Peng et al., 2024). Moreover, by optimizing network architectures and loss functions specific to the growth traits of fruit and vegetable flowers, and by incorporating attention mechanisms, researchers can enhance precision and real-time performance while minimizing computational demands.

For the computation of pollination poses, deploying an end-to-end deep detection network facilitates the identification of safe pollination zones, thereby preventing floral damage or rigid collisions (Eyles et al., 2022; Ge et al., 2019). Semantic segmentation and instance segmentation models are utilized to delineate the spatial and sequential data of pollination sites within complex backgrounds, enabling robots to execute precise and efficient pollination strategies (Ishita et al., 2020; Hao et al., 2023). Furthermore, geometric correlations between flowers and pedicels, derived from high-level semantic information, provide essential references for the structural design of end-effectors in pollination systems (Gené-Mola et al., 2020).

**Figure 3:**
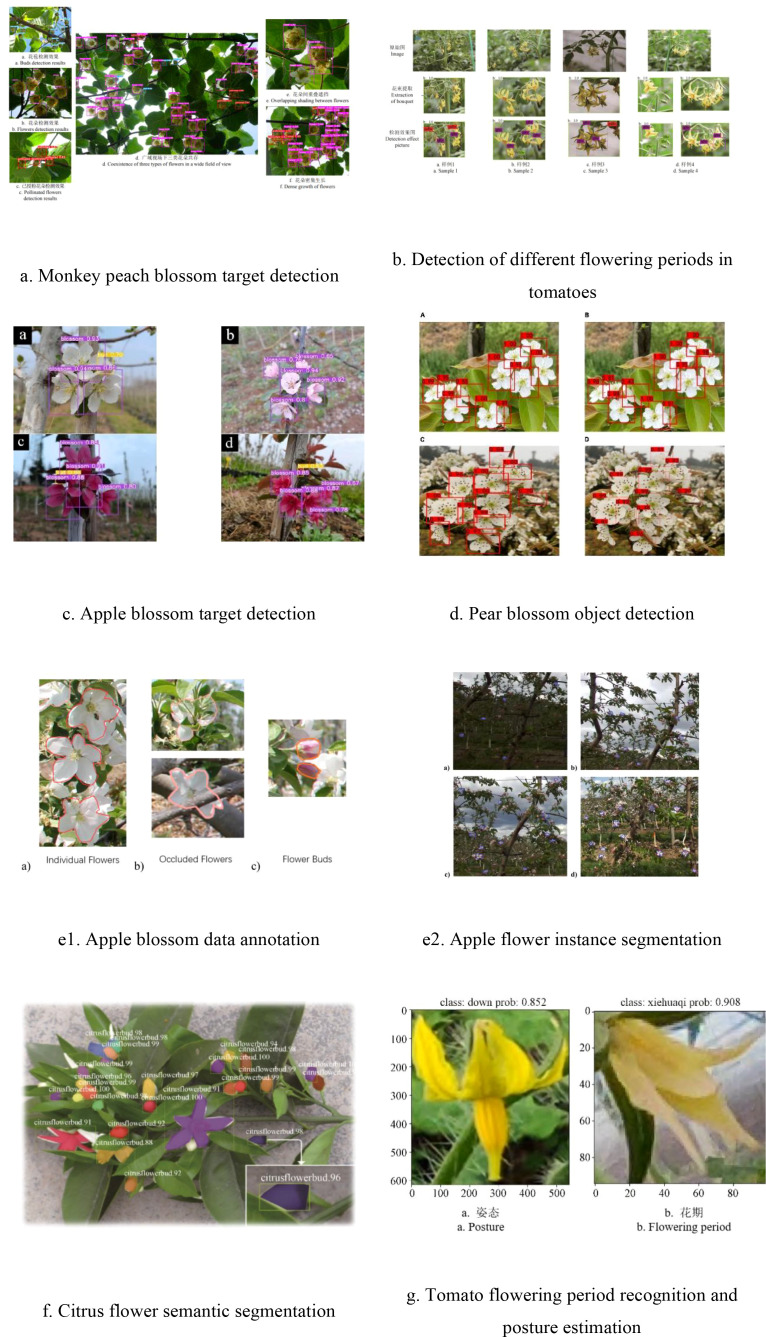
Schematic of fruit and vegetable flower object detection based on machine learning. **(a)** Monkey peach blossom target detection. **(b)** Detection of different flowering periods in tomatoes. **(c)** Apple blossom target detection. **(d)** Pear blossom object detection. **(e1)**. Apple blossom data annotation. **(e2)**. Apple flower instance segmentation. **(f)** Citrus flower semantic segmentation. **(g)** Tomato flowering period recognition and posture estimation.

In the realm of 3D reconstruction for pollination targets, utilizing advanced feature recognition via deep learning offers distinct advantages over the low-level feature detection of classical machine vision, particularly in improving the efficiency and stability of point cloud registration (Wang et al., 2019). Deep Generative Adversarial Networks (GANs) have proven effective in recovering missing surfaces in incomplete floral point clouds, thus enhancing reconstruction completeness and precision (Salim et al., 2023). Furthermore, since deep learning training necessitates substantial sample sizes, high-quality training datasets must encompass diverse perspectives, lighting conditions, scales, fruit shapes, and occlusion scenarios (Deng et al., 2020a), represented through various modalities including images, point clouds, voxels, and time series. To guarantee that network performance satisfies feature extraction criteria, it is imperative to conduct comprehensive deployment and testing in real-world scenarios (Chu et al., 2025; Deng et al., 2020). Beyond basic metrics such as accuracy and real-time performance, evaluating stability and scene adaptability is essential (Fan et al., 2023). **Figure 3** demonstrates the efficacy of machine learning in detecting fruit and vegetable flowers and estimating their poses, while **Table 1** provides a comprehensive summary of relevant research data.

**Table 1:** Recent research on machine learning-based fruit and vegetable flower object detection and pose estimation.

Serial number	Authors	For crops	Methods	For tasks
1	Deng et al (Deng et al., 2020)	Citrus flower	Mask R-CNN	instance segmentation
2	Fanet al (Fan et al., 2023)	honeysuckle	YOLOv5s+EfficientNet+ CARAFE	object detection
3	Gong et al (Gong et al., 2023)	macaque peach	YOLOv5s+C3HB+CCA	object detection
4	Si et al (Si et al., 2024)	Apple blossom	YOLOv5s + C-CoTCSP+ RFB+ VariFocal Loss	object detection
5	Mu et al (Mu et al., 2023)	Multiple flowers	SSD	object detection
6	Shang et al (Shang et al., 2022)	Apple blossom	YOLOv5s	object detection
7	Shang et al (Shang et al., 2022)	Apple blossom	YOLOv5s+ ShuffleNetv2 + Ghost Module	object detection
8	Qiu et al (Qiu et al., 2022)	Apple blossom	YOLOv4	object detection
9	Mu et al (Mu et al., 2023)	Apple blossom	RCNN	semantic segmentation
10	Chen et al (Chen et al., 2022)	Tomato flower	YOLOv5s+C2f_ScConv+ LSKA +ADown	object detection
11	Yue et al (Yue et al., 2024)	Soybean flower	YOLOv5+CA	object detection
12	Sebastian et al (Estrada et al., 2024)	peach blossom	YOLOv7x	object detection
13	Wang et al (Wang et al., 2022)	Ihwa	YOLOv4+ SENet + ShuffleNetv2	object detection
14	Xu et al (Xu et al., 2022)	Tomato flower	YOLOv3	object detection
15	Sun et al (Sun et al., 2021)	Apple, Peach, Pear Blossom	DeepLab-ResNet	semantic segmentation
16	Yu et al (Yu et al., 2022)	Tomato flower	YOLOv5s+EfficientNet	attitude estimation
17	Zhang et al (Zhang et al., 2024)	tomato	YOLOv5s	attitude estimation
18	Kuang et al. (Kuang et al., 2025a; 2025b)	Chili	YOLOv8n+YOLOv5n	object detection

### 2.3 Analysis, summary, and evaluation

The emergence of machine learning technologies in the field of agricultural visual perception has precipitated a paradigm shift in object detection applications, particularly for the identification of fruits, vegetables, and flowers. Through the continuous optimization of algorithmic architectures, the implementation of deep, multi-layered network designs, and rigorous training with specialized floral datasets, these methodologies have effectively surmounted challenges that baffled conventional techniques. These challenges include the accurate recognition of variable flower postures and the limitations in precision inherent to traditional image processing. Deep learning models exhibit remarkable robustness, stability, and applicability even when confronted with complex environmental disturbances, including dense foliage occlusion, variable illumination conditions, color overlap, and background noise. By leveraging potent feature extraction capabilities, these networks efficiently address multifaceted issues in agricultural vision and demonstrate significant potential for applications such as robotic pollination.

However, it is imperative to acknowledge that such sophisticated feature extraction mechanisms typically demand extensive annotated datasets and substantial computational power. In the context of facility agriculture, these resources are often constrained. Consequently, researchers are prioritizing the development of lightweight network architectures to alleviate the dependency on massive training data and high-performance computing hardware. Concurrently, advancements in data augmentation techniques are being continuously refined to enhance feature learning efficiency and model parameterization. These innovations facilitate the training of high-quality deep neural networks even under small-sample conditions, which is critical for the practical deployment of deep learning solutions in resource-constrained agricultural environments. In contrast to conventional target detection methods, deep learning-based approaches integrate algorithmic innovations with image preprocessing techniques to thoroughly extract high-dimensional semantic features of chili blossoms, thereby significantly elevating both recognition efficiency and accuracy.

## 3 Pollination mechanisms and end-effector design

### 3.1 Domestic and international pollination methods and their characteristics

Vision-based precise perception lays the foundation for efficient physical pollination, and end-effectors, as critical components that translate perceptual data into physical operations, have seen their design innovation and optimization become a central research focus. The rapid advancement of mobile robotics has extended its applicability across multiple dimensions of modern agriculture, establishing intelligent robots as a pivotal catalyst for enhancing agricultural automation. Within this context, pollination robots represent a critical solution, with research increasingly focusing on the innovation and optimization of their end-effector designs.

Current designs for fruit and vegetable pollination end-effectors are diverse, integrating various physical, biological, and chemical methodologies. Physical methods predominantly include spray-based techniques (Zou et al., 2023), which involve the direct application of pollinating media; vibration-assisted methods (Chechetka et al., 2017), which utilize mechanical oscillation to facilitate pollen dispersal; and contact-dipping approaches (Wu et al., 2008), which simulate manual pollination motions to apply pollen with precision. Specifically, spray-based techniques facilitate pollination through the direct application of the medium, vibration-assisted methods augment pollen distribution using mechanical vibrations, and contact-dipping methods replicate manual efforts to promote efficiency through precise application. These physical methods have demonstrated tangible efficacy in real-world applications and are continually refined as technology progresses.

In addition to physical approaches, biological methods may employ specific agents or mechanisms to enhance pollination, such as biomimetic strategies that imitate the natural behaviors of bumblebees (Yao et al., 2020). CChemical approaches may entail the use of substances designed to stimulate or potentiate the pollination process (Shimizu and Sato, 2018). These varied methodologies offer distinct technological avenues for improving pollination efficiency and success rates. When developing end-effectors for pollination robots, researchers must evaluate the structural principles and intended applications of these actuators, while considering their conventional use in various fruit and vegetable harvesting contexts. The design and performance specifications are inevitably influenced by the diversity of crop varieties and the complexity of unstructured agricultural environments. Consequently, the design of end-effectors necessitates a comprehensive evaluation of mechanical, electronic, material, and biological factors to ensure efficient, precise, and crop-safe operations. This section details the configuration of end-effectors for six types of facility-based mechanical pollination systems, analyzing and contrasting their operating principles, benefits, and drawbacks. The **Table 2** Mechanical devices for fruit and vegetable pollination, their applicable scenarios, and the crops they are designed for. And **Table 3** provides a comparative examination of these end-effector types.

**Table 2:** Mechanical devices for fruit and vegetable pollination, their applicable scenarios, and the crops they are designed for.

Pollination device	Applicable scenarios	For crops	End effector structure
rotor UAV pollination (Li et al., 2017; Zhang et al., 2021; Chechetka et al., 2017; Gao et al., 2023)	field	paddy	pneumatic
Blackberry Pollination Robot (Ohi et al., 2018)	greenhouse	blackberry	contact dipping
mimicry of pollination	greenhouse	tomato	pneumatic
buttercup pollination (Ding et al., 2014)	greenhouse	lily	bee mimic
Ultrasonic Pollination Robot (Li et al., 2014; Gao et al., 2023)	greenhouse	strawberry	ultrasonic
A Robot for Pollination of Kiwifruit (Ding et al., 2014)	orchard	Kiwifruit	atomizing
Air-fertilizer for kiwifruit (Kempe and Gils, 2011; Tacconi et al., 2016)	orchard	Kiwifruit	*pneumatic*
Kiwi Powder Sprayer (Dropcopter, 2022)	orchard	Kiwifruit	atomizing
Kiwifruit atomizer (Yuan et al., 2016)	orchard	Kiwifruit	*pneumatic*
Helicopter Pollinator (Yang et al., 2023)	field	paddy	atomizing
Tomato Hormone Pollination Robot (Wen et al., 2022)	greenhouse	tomato	atomizing
Flower of Forsythia suspensa mechanical arm pollination (Anon, 2022)	greenhouse	Forsythia suspensa Vahl	contact dipping
Tomato Pollination Manipulator (Li et al., 2026)	industrial factory	tomato	atomizing

**Table 3:** Structure of the end effector of the fruit and vegetable pollination robotic arm.

End effector structure	Principle	Advantage	Disadvantage
Airborne (Shimizu et al., 2015)	By intermittently blowing air into the flowers, the vibrations are induced to simulate natural wind or artificial shaking for pollination.	Prevent the asynchronous pollination of fruits and vegetables and save resources and environmental protection	incomplete pollination
spray type (Zou et al., 2023)	By mixing the pollen with water or other medium to form a suspension, which is then evenly sprayed onto the crop flowers using a sprayer, the natural pollination process is simulated.	Efficient and labor-saving	Polluting the environment, wasting the powder
mimicry of bumblebee flight (Yao et al., 2020)	The pollination process is accomplished by imitating the sharp sound of the hornet’s flight when collecting pollen to produce vibrations.	Environmentally friendly, labor-saving	high cost, pollen loss
Ultrasonic (Li et al., 2014)	The process of pollination is completed by the vibration of ultrasonic waves to make the pollen detach from the stamen and spread to the stigma of the pistil.	High pollination efficiency and no flower damage	High cost and easy to damage flowers
Oscillating ^(^Wang et al., 2013^)^	Pollination is achieved by using a vibrating device at a specific frequency to dislodge pollen from the stamen and disperse it to the stigma of the pistil.	Save labor, work Wide range, eco-friendly	Uneven pollination may cause flower damage
Contact dip	The precise pollination method involves transferring pollen to the stigma of the crop plant using physical tools such as brushes, cotton swabs, or small brushes.	Environmentally friendly and highly accurate	High labor intensity, low efficiency

### 3.2 Categories of pollination execution agencies

The safety and efficiency of agricultural pollination depend significantly on the synergy between apiculture and mechanical pollination technology. When selecting pollination methods, it is crucial to consider the biological traits of crops and their natural habitats. While natural wind typically suffices for self-pollinating crops, insect pollination is generally more efficacious for cross-pollinating crops or within confined environments. In the specific context of greenhouse pepper farming, where peppers are self-pollinating vegetables, the primary pollination methods include insects, wind, and manual techniques. Although each method possesses distinct advantages and constraints, all aim to improve pollination efficiency and success rates. Currently, manual pollination remains the predominant technique, whereas automated pollination acts as an essential adjunct when natural conditions are limited. Even under optimal natural conditions, the integration of apiculture and mechanical methods can optimize the process, with economic viability being a primary consideration. Consequently, the research and development of robotic pollination methods for *chili* are imperative. To address diverse pollination requirements, the development of specialized pollination machinery and end-effectors is essential. These devices must adeptly execute numerous functions, including accurately identifying pollen transfer locations, administering pollen by dipping and spreading, and facilitating pollen dissemination. The precise implementation of these actions is vital for guaranteeing superior pollination quality. **Table 2** delineates the research and application contexts for several fruit and vegetable crops regarding their suitable pollination techniques and end-effectors.

**Figure 4:**
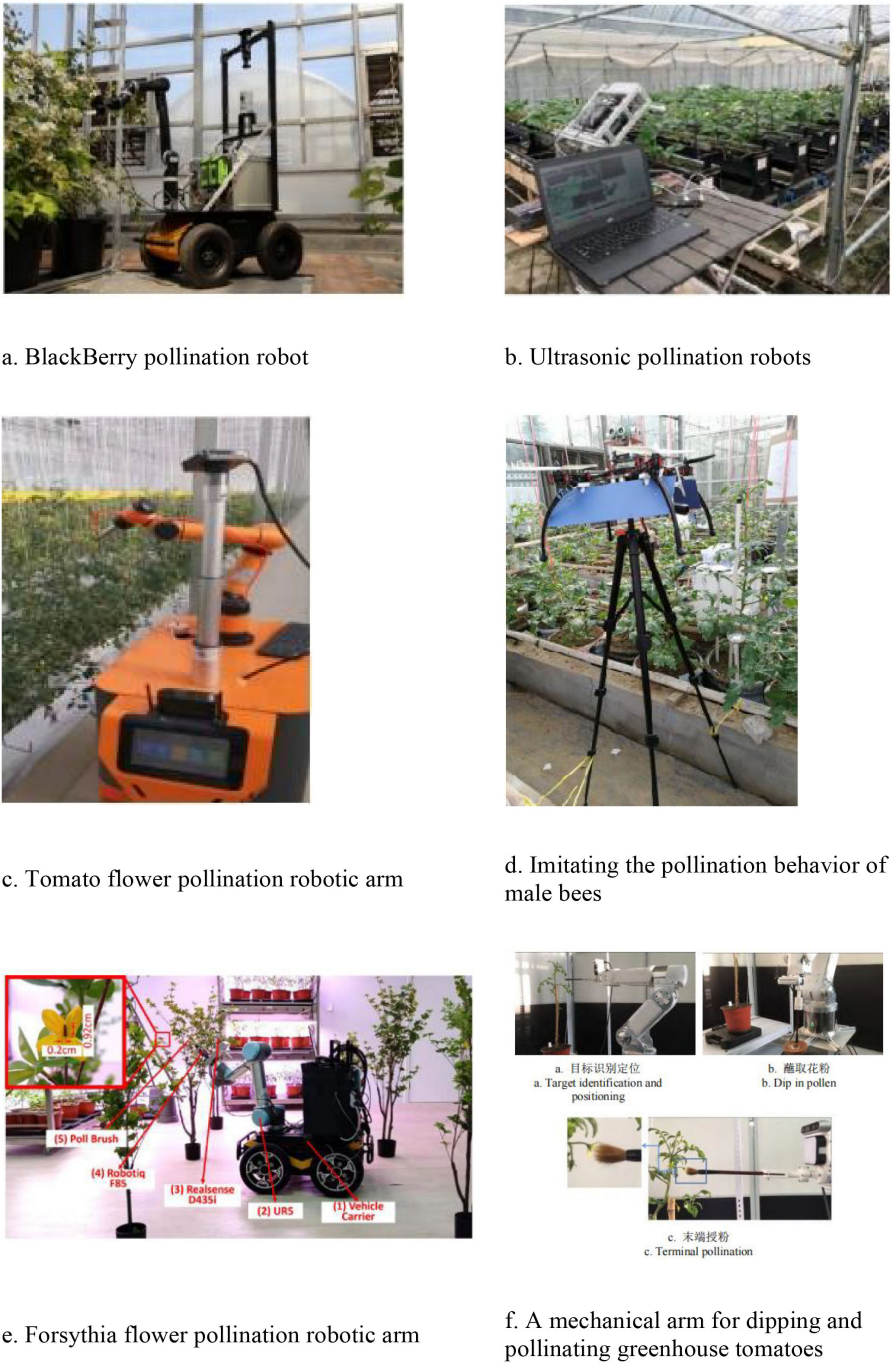
Schematic diagram of the effect of mechanical pollination device for greenhouse crops. **(a)** BlackBerry pollination robot. **(b)** Ultrasonic pollination robots. **(c)** Tomato flower pollination robotic arm. **(d)** Imitating the pollination behavior of male bees. **(e)** Forsythia flower pollination robotic arm. **(f)** A mechanical arm for dipping and pollinating greenhouse tomatoes.

**Figure 5:**
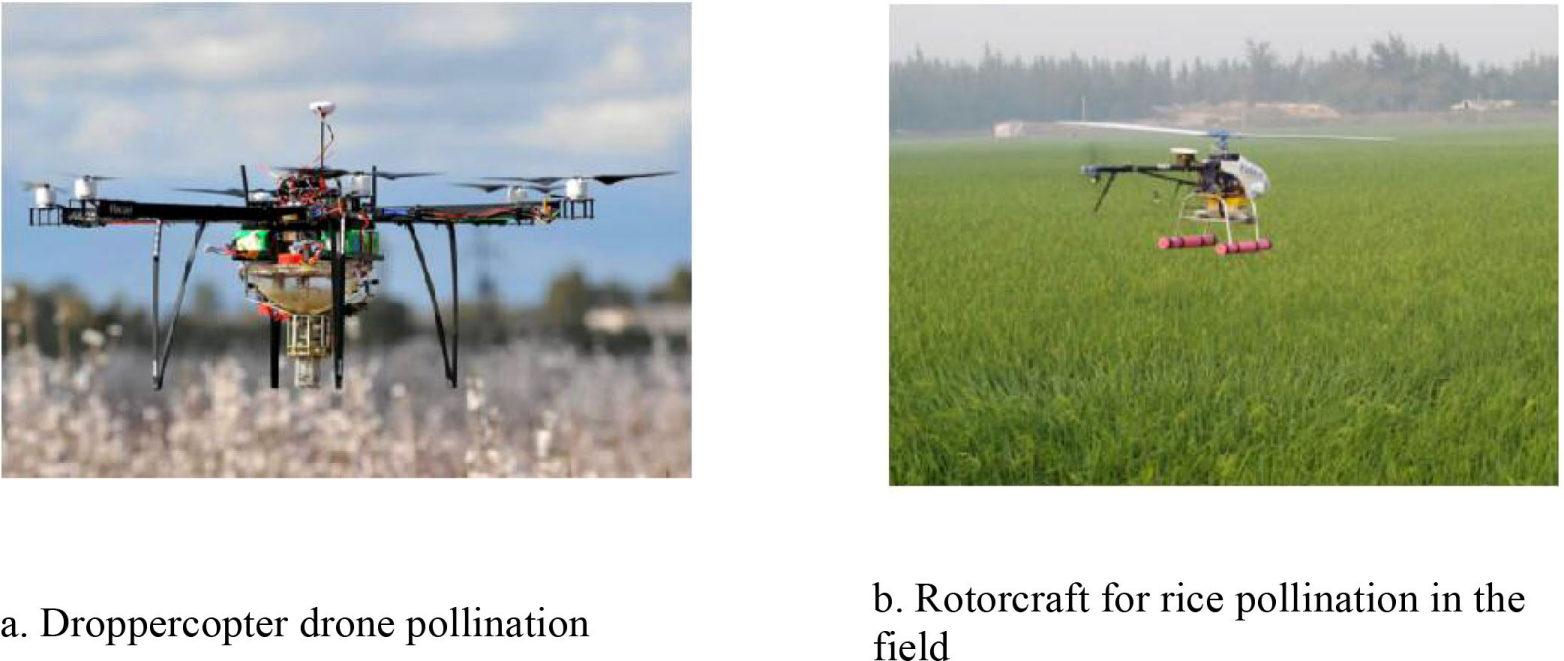
Schematic diagram of the effect of the field crop mechanical pollination device. **(a)** Dropper copter drone pollination. **(b)** Rotorcraft for rice pollination in the field.

**Figure 6:**
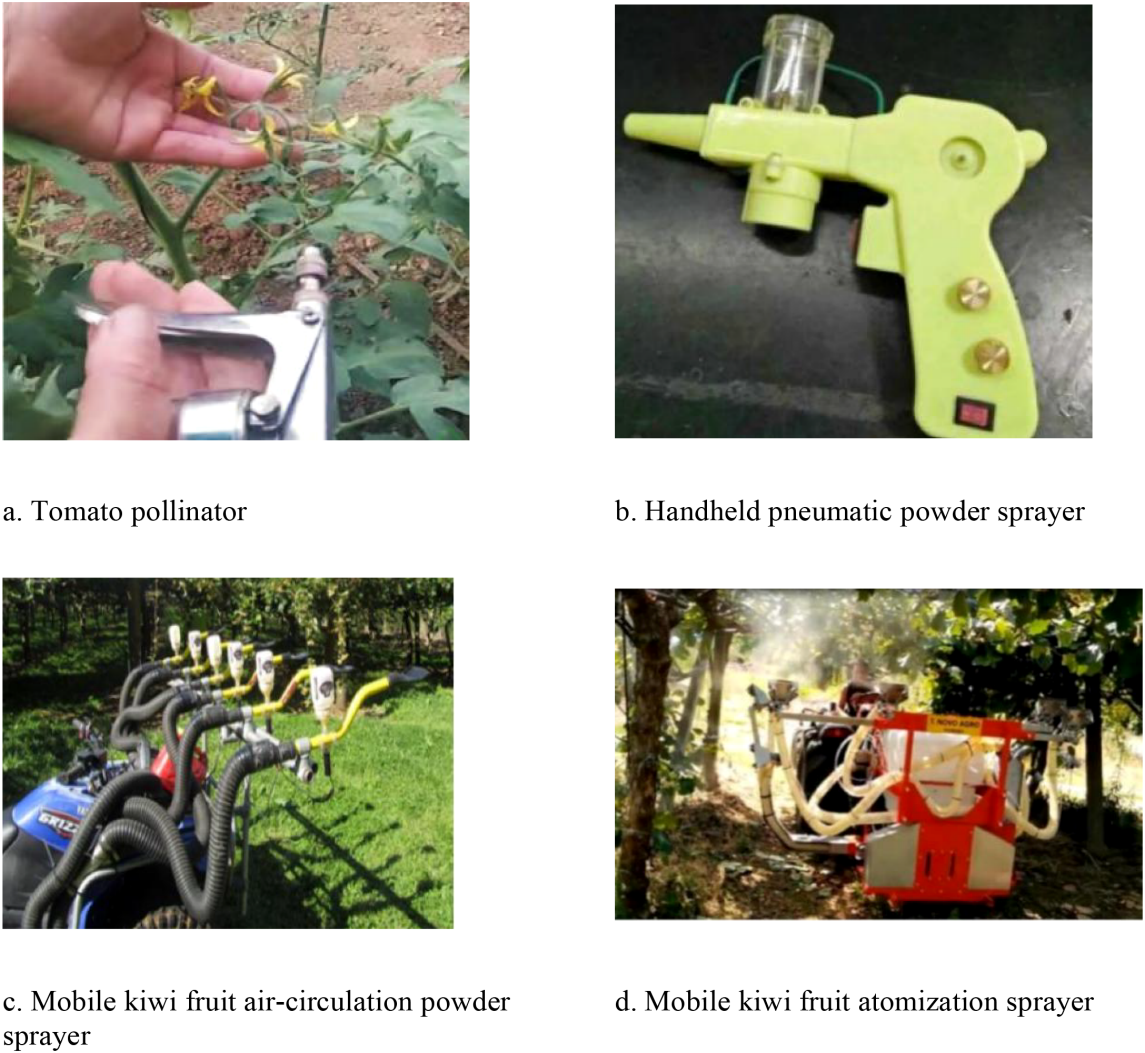
Schematic diagram of the effect of mechanical pollination device for orchard crops. **(a)** Tomato pollinator. **(b)** Handheld pneumatic powder sprayer. **(c)** Mobile kiwi fruit air-circulation powder sprayer. **(d)** Mobile kiwi fruit atomization sprayer.

As illustrated in **Table 2**, robotic pollination in field crops generally utilizes pneumatic-assisted methods to replicate natural wind forces (Chang et al., 2023; Suming et al., 2015). However, for orchard crops, employing rotary-wing aircraft to simulate wind-assisted pollination is often unfeasible due to extensive tree canopies and broad planting intervals. The asynchronous flowering cycles of many fruit tree species further complicate this process. To draw analogies and demonstrate potential technologies that can be adapted for pepper pollination For cross-pollinating crops such as apples, pears, and kiwifruits, where ample pollen can be harvested and preserved, air-blowing or atomization methods are typically employed. In greenhouses, where spatial constraints exist, pollination apparatus must be engineered to be compact and miniaturized. **Figures 4**–**6** depict machine learning-based solutions for various pollination scenarios and devices for fruit and vegetable blossoms, respectively.

### 3.3 Analysis, summary and evaluation

A comprehensive examination of advancements in domestic and international pollination machinery and end-effector designs reveals several significant findings and future opportunities. Contemporary pollination devices exhibit exceptional ingenuity and adaptability through the integration of physical, biological, and chemical methodologies. The incorporation of deep learning into agricultural visual perception, alongside progress in mechanical pollination technology, has markedly improved the intelligence and precision of pollination robots. Nevertheless, these systems continue to encounter obstacles regarding flexibility, accuracy, and cost-effectiveness, particularly within intricate and dynamic agricultural settings.

Future research should concentrate on augmenting the intelligence of pollination equipment by expanding environmental awareness, optimizing decision-making processes, and upgrading adaptive control systems. Concurrently, it is essential to investigate more efficient and cost-effective pollination technologies, such as the integration of artificial intelligence with natural pollination methods to enhance both efficiency and quality. Moreover, with the advancement of sustainable and precision agriculture, research on pollination machinery must consider environmental impacts and resource efficiency to promote the ecological transformation of agricultural production. Through interdisciplinary collaboration and technological innovation, the field can promote extensive application and widespread adoption of advanced pollination machinery (Jiyu et al., 2017; Shang et al., 2024).

## 4 Research progress on pollination strategies and motion control

### 4.1 Pollination strategies

An effective and adaptable pollination control strategy is essential for addressing the complex sequence planning required for mobile pollination robots in greenhouse pepper cultivation. By utilizing machine learning algorithms to identify and locate various target flowers and their orientations, it becomes possible to determine the precise centroid of each blossom. Consequently, an optimized pollination strategy can be devised, enabling the generation of optimal trajectory paths (Ni et al., 2025).

Ohi et al. (2018) developed a robot named BrambleBee to address pollination challenges in thorny vegetation. This system integrated technologies for recognition, trajectory planning, and motion regulation. Building upon BrambleBee, the team subsequently introduced the six-arm pollinating robot Stickbug in 2022, which significantly improved pollination efficiency. Similarly, Yuan et al. (2016) developed a tomato pollination robot utilizing a four-degree-of-freedom (4-DOF) robotic arm, achieving accurate detection and collision-free motion control. Gao et al. (2023) engineered a kiwifruit pollination robot featuring an end-effector equipped with a nozzle and a recovery mechanism, which markedly reduced pollen loss. In this context, redundant robotic arms have emerged as a significant area of research due to their proficiency in obstacle avoidance within intricate environments. Baur et al. (2012) engineered a modular redundant robotic arm capable of versatile mobility. In a 2023 study, Colucci et al. (2023) decoupled the motion of redundant robots by treating the mobile chassis as a distinct degree of freedom. Furthermore, Schuetz et al. (2015) proposed a unified planning approach for redundant degrees of freedom in robotic arms, facilitating advancements in obstacle avoidance for complex agricultural settings (Wu and Fang, 2025; Tao et al., 2024).

**Figure 7:**
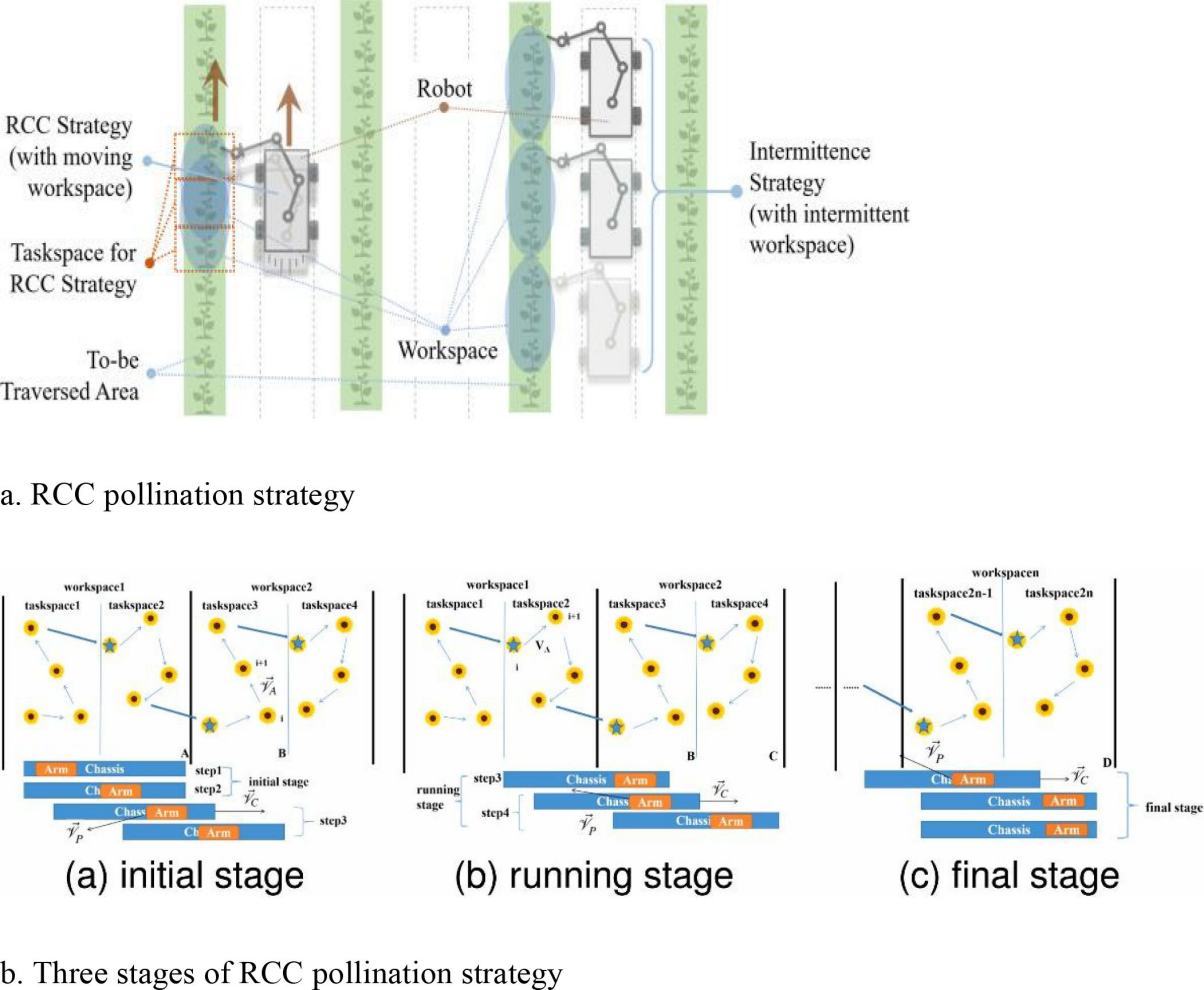
RCC pollination strategy and its three stages. **(a)** RCC pollination strategy; **(b)** three stages of RCC pollination strategy, including (a) initial stage, (b) running stage, and (c) final stage.

To optimize efficiency and guarantee high success rates, pollination robots must traverse all target flowers via the most direct route. This necessitates the ability to recognize flowers within the visual field, strategically devise efficient paths, and synchronize robotic arm movements. Wei et al. (2024) introduced an innovative Redundant Cooperative Control (RCC) strategy to address challenges associated with conventional greenhouse tomato pollination, such as high labor costs, environmental concerns regarding chemical use, and fluctuating bee populations. The RCC technique partitions the robot’s workspace into subspaces, solves the Traveling Salesman Problem (TSP) for each zone, and synchronizes end-effector and redundant movements to ensure accurate task transitions. As depicted in **Figure 7**, the methodology encompasses one-dimensional and multi-dimensional RCC techniques, outlining the initial, operational, and final phases of path planning and execution. By leveraging the redundant motion capabilities of both the chassis and the arm, this strategy significantly enhances efficiency. Validation studies on a pollination robot platform demonstrated an average rate of 7.5 seconds per flower, representing a 36.4% improvement over prevalent intermittent strategies.

### 4.2 Path planning and motion control

Motion control is a cornerstone of robotic pollination, incorporating sophisticated technologies including machine learning, path planning, and kinematic control to facilitate autonomous positioning and accurate operation in unstructured environments like greenhouses. This process simulates manual pollination motions to generate robotic arm trajectories and force control profiles, ensuring both operational efficiency and high success rates.

**Figure 8:**
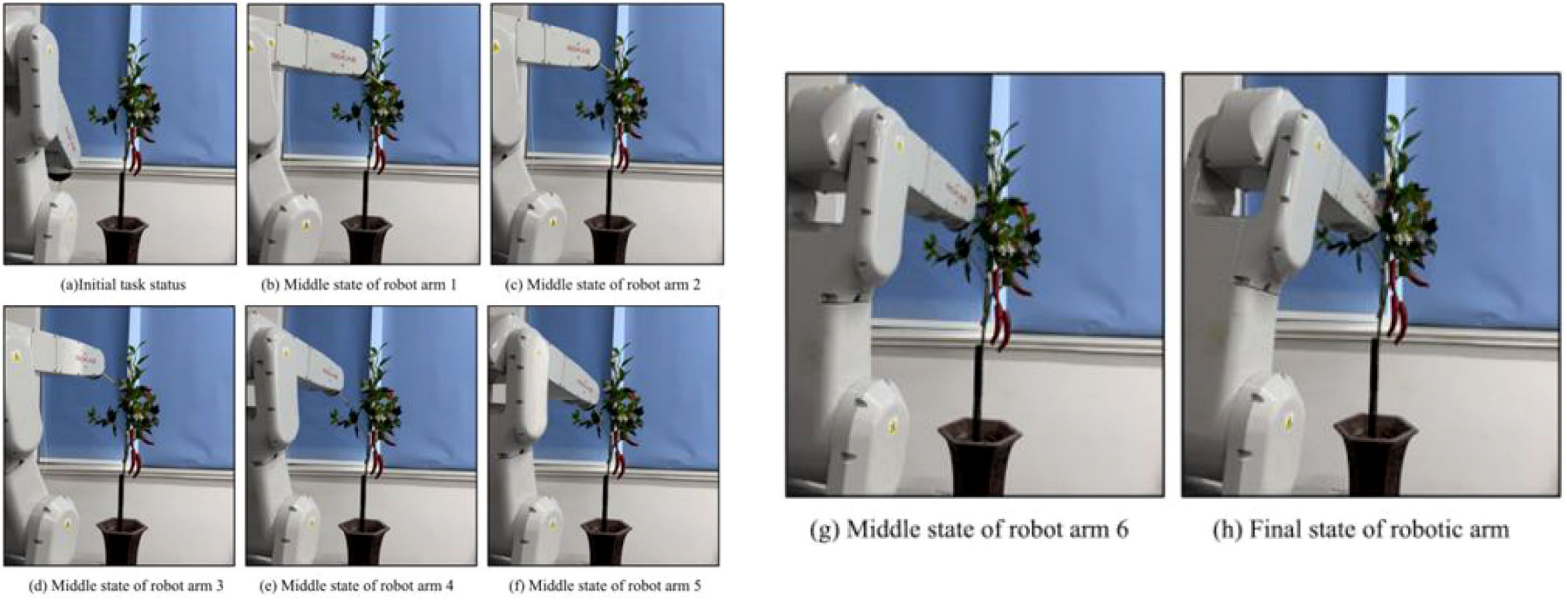
Path planning and obstacle-avoidance motion control of pepper flower pollination manipulator: **(a)** Initial task status; **(b–g)** middle states of the robotic arm during the movement process (from stage 1 to 6); **(h)** final state of the robotic arm.

Li et al. (2022) developed a novel ground robot to serve as an autonomous pollinator for kiwifruit orchards, verifying its performance under real field conditions. The study utilized the YOLOv4 algorithm alongside transfer learning to achieve precise identification of kiwifruit flowers and buds (Li et al., 2022a; 2022b; Lin et al., 2023;Ahmad et al., 2024). In subsequent experiments, Li et al. (2025; 2026)  innovatively assessed flower opening orientation via center-of-gravity measurement. This advancement enabled the precise positioning of robotic arms, ensuring accurate alignment with the pistils and petal curves. Conversely, Strader et al. (2019) developed the BrambleBee unmanned ground vehicle, which focused on specific flower orientations rather than encompassing all potential directional states. To address the need for automated pollination amidst declining bee populations, Khubaib et al. (2021) introduced a vision-guided servo control technique. As illustrated in **Figure 8**, this method uses deep learning to detect bloom size and orientation, converting these metrics into depth data to drive a visual servo platform. A six-degree-of-freedom (6-DOF) control system is then utilized to execute the pollination movements with high precision.

**Figure 9:**
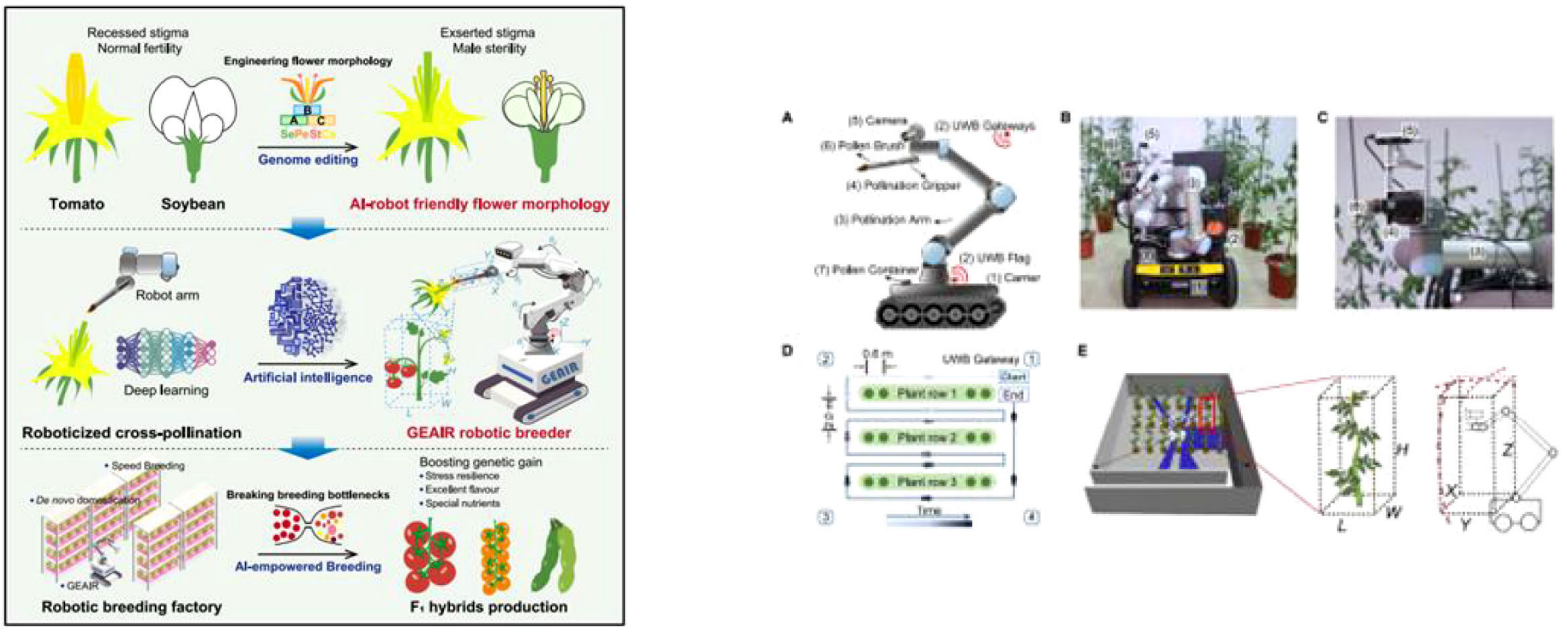
Collaborative design strategy of tomato robot pollination based on genome editing and artificial intelligence robot synergy. The left panel illustrates the integrated breeding and pollination pipeline. The right panel presents the robotic system and navigation details: **(A)** key components of the GEAIR robotic pollinator, including (1) carrier, (2) UWB gateways/flag, (3) pollination arm, (4) pollination gripper, (5) camera, (6) pollen brush, and (7) pollen container; **(B, C)** photos of the robot operating in the greenhouse environment; **(D, E)** schematic diagrams of the UWB-based navigation path and greenhouse layout.

Artificial intelligence (AI) and robotics offer substantial prospects for advancing precision agriculture. However, intrinsic crop characteristics, such as floral structures with concave stigmas, limit the application of robots in breeding processes. Xie et al. (2025) introduced a crop-robot co-design strategy termed “Genome Editing and AI Robotics” (GEAIR) to overcome this bottleneck. This approach involves creating male-sterile tomato lines with exposed stigmas via genome editing, paired with mobile robots trained for automated stigma recognition. The system, depicted in **Figure 9**, achieved efficiency comparable to manual techniques in F1 hybrid breeding. This strategy not only validates automated hybrid breeding but also demonstrates the potential of merging AI with gene editing to accelerate the development of climate-resilient crops.

### 4.3 Analysis, summary and evaluation

The ongoing agricultural technology revolution has introduced innovative and effective pollination systems for fruit trees, such as kiwifruit. Selective flower recognition and liquid pollination technology have emerged as key achievements, markedly improving pollination accuracy and efficiency via precision intermittent spraying tactics (Liu et al., 2023). Nonetheless, current fruit and vegetable pollination robots encounter obstacles, including their cumbersome dimensions and limitations in seamless integration inside protected agricultural settings. Concurrently, the swift advancement of household spray technology presents viable remedies to this problem (Ma et al., 2025; Oubounyt et al., 2019; Ferreira et al., 2023). Ahmad et al. (2024) developed accurate and robust pollination methods for watermelons using intelligence-guided visual serving, which is a sophisticated motion control strategy (Li et al. (2025); Li et al., 2026). focused on the design and performance verification of an intelligent pollination robot specifically for facility tomatoes, inherently addressing the motion and control required for the task. Hao et al. (2026) created a clip-assisted flower detection and wind-compensated precision liquid pollination robot for kiwifruit orchards, directly incorporating a compensation strategy into the motion control system to handle environmental disturbances. Furthermore, Liu et al. (2023) contributed to the upstream perception required for control by researching tomato flower pollination features recognition based on binocular gray value-deformation coupled template matching, which is essential for guiding the robot’s motion (Zhang et al. (2025); Akdoğan et al., 2025). provided a comprehensive review of autonomous flower pollination techniques, discussing the progress, challenges, and future directions of various control and strategy methodologies. While many papers concentrate on detection, such as Li et al. (2022) work on deep learning-based kiwifruit flower recognition, and Li G. et al.’s real-time detection of kiwifruit flower and bud using YOLOv4, these perception capabilities form the indispensable foundation for the robot’s subsequent motion planning and strategic execution during the pollination process. Broader contextual control is also evident (Jiang and Ahamed, 2025), as seen in Chen A (Chen and Huang, 2025). and Huang et al. (2025) work on integrating reinforcement learning and large language models for crop production process management optimization and **control. Conversely,** the pepper sector predominantly depends on conventional hand powder-dipping techniques for pollination, which are ineffective and expensive. To resolve this, we can investigate the integration of kiwifruit’s spray pollination theory with manual pollination methods for tomatoes. By examining the distinct development traits and pollination needs of crops such as peppers and tomatoes, we can enhance flower target detection algorithms for improved pollination management. The implementation of a redundant collaborative control variable spraying approach utilizing RCC (Redundant Collaborative Control) at pepper stamens facilitates precise, variable spraying that optimizes pollination while markedly diminishing chemical residues and pollen waste. This method exhibits superior environmental efficacy and ensures accurate pollination for peppers.

## 5 Current challenges and solutions

Pollination technology, serving as a pivotal auxiliary innovation facilitating the advancement of simplified facility agriculture, has remained nascent and constrained within China’s greenhouse pepper industry. Currently, the majority of regions depend primarily on conventional manual pollination techniques, supplemented by natural vectors such as bees and wind. This reliance is problematic due to seasonal fluctuations, intricate internal facility conditions, and elevated labor costs and intensity, all of which hinder the intelligent transformation of greenhouse pepper pollination. Contemporary research has largely concentrated on accurate flower identification and pose estimation for various fruit and vegetable crops, aiming to improve the detection precision of pollination targets. However, there is a distinct lack of research regarding the design of mechanical pollination end-effectors and control strategies specifically for chili.

Efficient pollination management solutions are required to resolve the sequencing challenges faced by mobile pollination robots in greenhouse pepper cultivation. These strategies must employ machine vision recognition algorithms to identify and locate various target flowers and their orientations, ascertain the centroids of pollination targets, and devise optimal path planning for efficient operation. Furthermore, the design of end-effectors is essential for enhancing operational precision and adaptability. Precise mechanical design and sophisticated control algorithms enable gentle contact and accurate pollination of pepper flowers without inflicting damage. The precision and rationality of these elements directly influence the accuracy and operational efficiency of the pollination machinery.

In the domain of facility agriculture, the exact identification, classification, and pose estimation of chili flower buds, as well as the precise positioning and regulation of pollination mechanisms, constitute significant hurdles in the progression of robotic precision pollination technology (Xu et al., 2023; Zhang et al., 2024; Liu et al., 2023). Investigating precision pollination approaches for chili in controlled agricultural environments possesses substantial practical significance for enhancing seed production technology and offering essential theoretical support. A comprehensive examination of these technologies is crucial for fostering innovation and advancement in chili seed production.

### 5.1 Current challenges

The viability of essential pollination technologies for chili peppers necessitates further investigation and refinement. The pollination process requires exceptional accuracy and sensitivity, encompassing precise flower identification, effective pollen transmission, and successful pollination outcomes. These requirements present considerable hurdles for flower target detection and pose estimation algorithms. Potential difficulties include false positives and missed detections resulting from low recognition accuracy, as well as diminished pollination efficiency attributable to inadequate precision. Furthermore, design deficiencies in recognition algorithms and end-effectors for pollination robotic arms often precipitate a cascade of issues, either directly or indirectly. These issues result in heightened pollen loss during successive pollination events and may inflict unnecessary physical harm to chili plants, thereby negatively impacting yield and quality. Consequently, ongoing enhancement and innovation in flower recognition algorithms and end-effector design are essential for improving overall pollination efficacy and practical application effectiveness. Ultimately, pollination strategies are crucial for the mechanical pollination of chili peppers, as they influence success rates and consistency while directly impacting seed quality and yield. Efficient pollination tactics can optimize path planning, potentially doubling results with reduced effort while conserving time and augmenting total equipment efficacy. Existing major methods for facility-based chili pollination continue to encounter the following obstacles:

#### 5.1.1 Flower recognition and pose estimation: limited robustness in adverse lighting and occlusion scenarios

Current techniques for detecting fertilized flowers predominantly depend on color image data obtained from binocular depth vision systems. These approaches extract and identify pepper flowers by assessing saturation, hue, chromatic attributes, and dimensional data. They utilize stereo vision technology to ascertain the 3D spatial coordinates of target flowers for positioning. However, in the intricate and variable production environments of contemporary agricultural facilities, existing recognition algorithms for pepper pollination encounter severe constraints under fluctuating lighting and occlusion conditions. This results in generally poor recognition rates (typically around 80% accuracy), frequent false positives, and missed detections, highlighting the necessity for improved robustness. Additionally, the low frame rate of detection processes leads to reduced processing speeds, extending the entire pollination workflow. This inefficiency fails to satisfy contemporary agricultural requirements for efficient and mechanized pollination, thereby limiting extensive adoption in large-scale production.

#### 5.1.2 Waste of pollen resources and operational inefficiency

The efficacy of pepper pollination relies on both precise dosage management and the configuration of the pollination apparatus. Current spray and contact pollination systems partially fulfill the fundamental criteria for intelligent pollination and effectively tackle the issue of asynchronous flowering on the same plant. However, they inadequately regulate pollen application rates, resulting in resource inefficiency. Furthermore, the comparatively slow operation speed of current end-effectors extends the entire procedure, diminishing overall efficiency. Although the initial investment in robotic pollination systems is high, the rising labor costs in rural China and the aging of the agricultural workforce are rapidly reducing the economic viability of manual pollination. Therefore, the long-term adoption of robotic pollination has strategic advantages; by achieving continuous operation and standardized precision, these systems can significantly reduce unit seed production costs and mitigate the risks associated with seasonal labor shortages.

#### 5.1.3 Restricted applicability and lack of versatility

A deficiency in adaptability continues to be a significant obstacle in the advancement of agricultural machinery. Numerous current designs are restricted to specific environmental conditions or crop varieties, thereby constraining the utility of end-effectors. Due to the varied pollination mechanisms and morphological traits of different plants, contemporary pollination robots frequently encounter difficulties in adapting to changes in working conditions or target crops, leading to diminished efficiency or total failure. Pepper pollination robots in facilities encounter comparable constraints in adaptation. Nonetheless, ongoing developments in science and technology are anticipated to yield increasingly versatile pollination robots in the future. These advanced devices will adapt to various working situations and crop needs, providing more efficient and convenient pollination options for agricultural production.

#### 5.1.4 Reliance on manual pollination techniques

Research on intelligent pollination systems for greenhouse peppers in China is presently in its nascent stages. The design of end-effectors primarily concentrates on two categories: spray (pneumatic) systems and contact dip techniques. Despite notable advancements in spray pollination technology across other domestic sectors, this sophisticated method has not been extensively implemented for greenhouse pepper pollination. Pepper pollination robots encounter several technical challenges and practical obstacles in real-world applications, including the need to enhance recognition accuracy, optimize pollination efficiency, improve environmental adaptability, and increase versatility. Consequently, to effectively address the intricate requirements of the current planting environment, it remains essential to persistently enhance research and development initiatives, refine pertinent technologies, and facilitate the maturation and dissemination of mechanical pollination technology for chili peppers.

### 5.2 Solution strategies and methods

The primary and most critical objective of mechanical pepper pollination is the accurate detection of targets and the estimation of flower poses. Consequently, it is imperative to construct a comprehensive and diverse dataset for pepper flower detection and pose estimation. This data foundation must thoroughly encompass images of pepper blossoms across various growth phases, lighting conditions, angles, and occlusion scenarios to guarantee the dataset’s comprehensiveness and representativeness. Based on these dataset characteristics, high-performance target detection algorithms, such as Convolutional Neural Networks (e.g., YOLO) or their enhanced deep learning variants, should be meticulously selected and trained to achieve accurate identification and pose estimation. This approach will elevate detection accuracy and improve the algorithm’s adaptability and robustness to complex environmental changes. The proposed device will incorporate several advanced technologies to create a scientifically robust and efficient operational system. It will utilize sophisticated machine learning methodologies for the intelligent recognition and assessment of pepper growth status; implement optimized Redundant Collaborative Control (RCC) pollination strategies to ensure precision and efficiency; integrate advanced path planning algorithms to facilitate autonomous navigation in intricate agricultural settings, avoiding collisions and optimizing pollination routes; and combine high-precision motion control technology to achieve seamless and accurate pollination operations.

Secondly, a customized mechanical pollination apparatus for chili peppers must be designed by amalgamating agricultural technology with crop-specific growth patterns. Despite China’s considerable advancements in liquid atomization research through thorough investigation of spray pollination technology, its application in greenhouse pepper cultivation remains nascent. Although spray machinery technology continues to develop, practical application encounters several challenges, including structural incompatibility with pepper flower anatomy, incongruity between spray droplet size and pollination needs, and difficulties in achieving precise timing control. These constraints impede the extensive implementation of spray technology in liquid pollination processes, especially in greenhouse agriculture where such technology is notably deficient, underscoring the pressing necessity for technological advancement. The proposed device will utilize advanced machine learning techniques via sophisticated image recognition and processing abilities to ensure precise flower identification and localization. It will also integrate RCC pollination methodologies to guarantee precision and efficacy. The technology utilizes advanced path planning algorithms to autonomously optimize pollination paths according to field or greenhouse layouts, thereby reducing redundant tasks and enhancing efficiency. Motion control technology ensures consistent performance in intricate surroundings, sustaining precise operations in both flat farms and structurally complex greenhouses.

**Figure 10:**
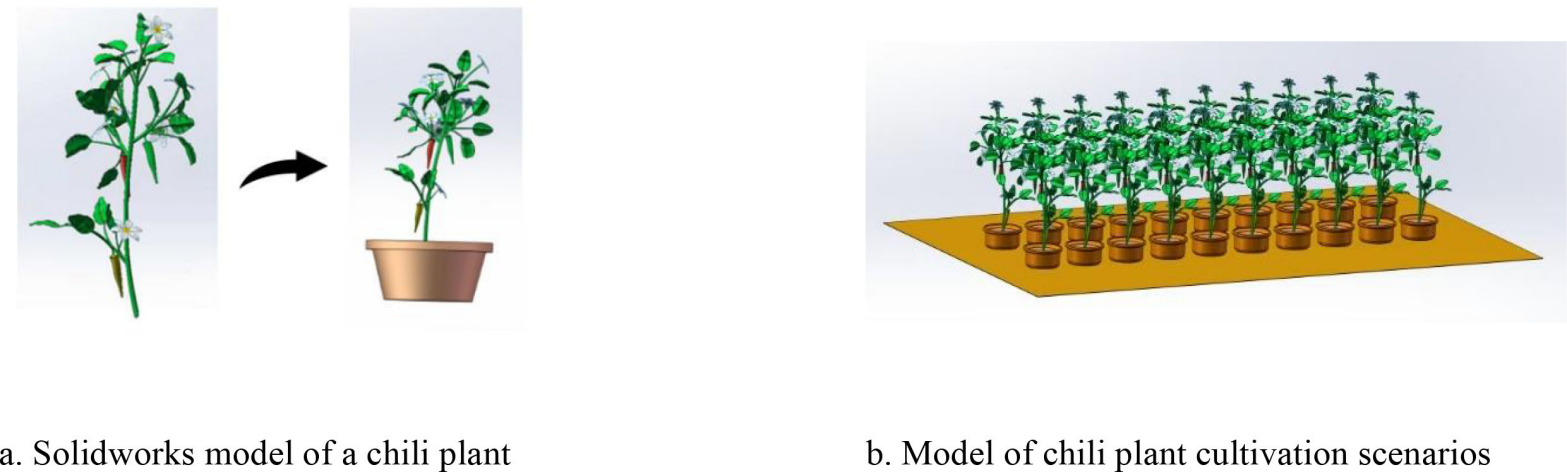
Facility pepper plant and planting scenario model. **(a)** Solidworks model of a chili plant. **(b)** Model of chili plant cultivation scenarios.

**Figure 11:**
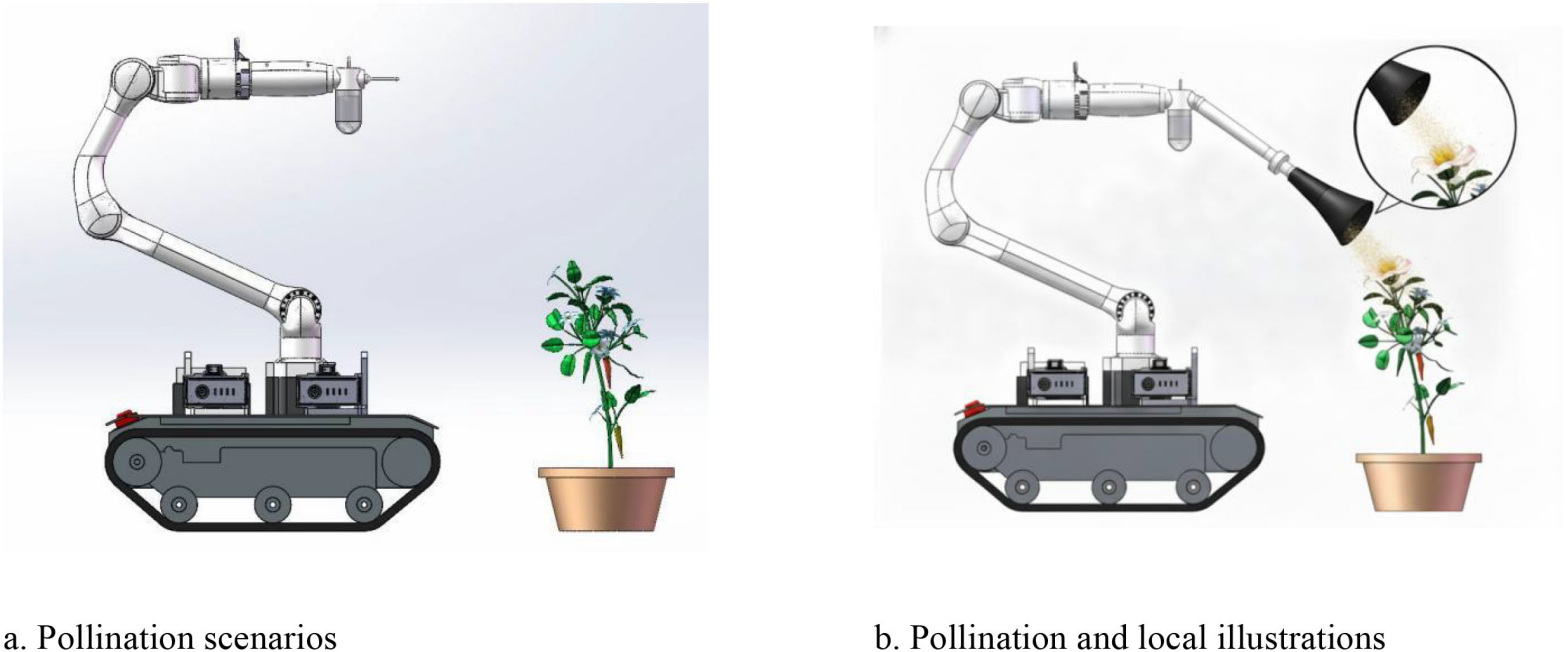
Application scenarios for pepper pollination using facilities. **(a)** Pollination scenarios. **(b)** Pollination and local illustrations.

To address these issues, this study leveraged the growth characteristics of greenhouse pepper plants. As illustrated in **Figure 10**, SolidWorks was employed to create a model of a pepper plant (**Figure 10a**) and a model of a cultivation scenario (**Figure 11b**). Furthermore, as shown in **Figure 11**, the incorporation of a spray-based pollination end-effector with a six-axis robotic arm facilitates the accurate pollination of pepper plants. This innovative method, integrating modern spray technology with precision robotic arms, aims to improve pollination efficiency and uniformity while decreasing labor intensity and economic costs, providing a novel solution for pepper pollination in greenhouse agriculture.

## 6 Summary and outlook

Looking ahead, there is compelling evidence to anticipate that mechanized precision pollination and seed production technologies will achieve widespread implementation across a broader spectrum of crop species. The synergistic integration of deep learning with pose estimation algorithms presents immense potential and value for the detection of crop flowers. By leveraging the robust feature extraction capabilities of deep learning alongside the precise spatial and directional predictions provided by pose estimation, it is possible to significantly elevate the levels of automation and intelligence in agriculture. This is particularly relevant for critical operations such as flower pollination and fruit harvesting. Such breakthroughs not only contribute to safeguarding global vegetable production and fostering sustainable agricultural development but also serve as pivotal technologies propelling the modernization of the agricultural sector.

The evolution of crop pollination apparatus and methodologies is progressively moving toward intelligence and precision to mitigate the challenges posed by declining natural pollinator populations. Future advancements in this domain will integrate artificial intelligence, machine learning, and robotics to drive innovative transformations within the industry. The development and deployment of these technologies are poised to exert a profound influence on the sustainable growth of global agriculture.

To augment the coordination efficiency within the workflow encompassing perception, decision-making, and execution for pollination robots, researchers must focus on two critical areas. First, it is essential to conduct rigorous analyses of the distribution characteristics of target objects within agricultural facilities. Incorporating these attributes as supplementary constraints into algorithms for visual identification, path planning, and motion control will facilitate the creation of specialized algorithms tailored to pollination environments. This approach optimizes the coordination mechanisms between the robot’s perception, decision-making, and execution modules. Second, a comprehensive performance evaluation framework must be established to assess the continuous operational state of robotic arms in orchard or greenhouse settings. This system should not only evaluate individual modules, such as visual systems, path planning, and navigation, but also formulate comprehensive metrics that assess the entire perception, decision, and execution workflow. These metrics will quantify the synergistic effects between modules and the overall operational efficacy of the robot, thereby meeting future requirements for extensive system testing. These measures will assist researchers in developing autonomous robotic systems that exhibit superior performance, reduced costs, and greater applicability for agricultural practitioners.

Addressing the aforementioned technical challenges is paramount for the advancement of research on precision pollination technologies for greenhouse peppers. Resolving these issues is of critical importance for promoting the practical application of pepper flower detection, pose estimation, and end-effector technologies, ultimately elevating the intelligence level of facility-based pepper pollination.
